# Identifying a novel variant in *ELOC* (*TCEB1*)*-*mutant renal cell carcinoma: a case report and literature review

**DOI:** 10.3389/fonc.2026.1989742

**Published:** 2026-10-21

**Authors:** Yushan Wang, Xuefei Ning, Guofu Xiao, Sumei Wu, Wen Guo, Junfeng Guo, Cheng Lei, Songqing Fan, Xinxue Ma

**Affiliations:** 1 Department of Pathology, People’s Hospital of Longhui County, Shaoyang, Hunan, China; 2 Department of Pathology, The Second Xiangya Hospital, Central South University, Changsha, Hunan, China

**Keywords:** *ELOC*, genetic testing, novel mutant variant, renal cell carcinoma, *TCEB1*

## Abstract

**Background:**

*ELOC* (*TCEB1*)-mutant renal cell carcinoma (RCC) is a recently recognized molecular subtype formally included the 2022 World Health Organization (WHO) classification. Although morphologically overlapping with clear cell renal cell carcinoma (ccRCC), it remains clinically and radiographically indistinguishable from other RCC subtypes. Because few cases have been reported, we describe an *ELOC (TCEB1)*-mutant RCC harboring a novel somatic *TCEB1* variant, c.227A>G (p.Y76C).

**Case presentation:**

A 52-year-old male presented with an incidentally discovered left renal mass on routine ultrasound. Computed tomography (CT) revealed a round, slightly hypodense lesion, approximately 14×15 mm, in the left kidney. Following retroperitoneal laparoscopic partial nephrectomy, pathological examination and immunohistochemistry (IHC) established the diagnosis, and next-generation sequencing (NGS) identified a novel *ELOC* (*TCEB1*) mutation, c.227A>G (p.Y76C).

**Conclusion:**

We report a rare *ELOC (TCEB1)*‐mutant RCC carrying one novel missense variant, c.227A>G (p.Y76C), and a frameshift variant, c.171_172insCC (p.N58Pfs*48), in trans. This case adds a newly identified *TCEB1* variant to the known spectrum of *ELOC*-mutant RCC and may help clinicians recognize this rare subtype. We also review the clinicopathological features, immunophenotype, molecular genetic alterations, differential diagnosis, treatment, and prognosis of this entity.

## 1 Introduction

*ELOC* (*TCEB1*), localized at 8q21, encodes Elongin C protein, an essential subunit of the von Hippel-Lindau (VHL) E3-ubiquitin ligase complex (1). As a core component, pVHL mediates ubiquitin-dependent degradation of hypoxia-inducible factor-α (HIF-α) (2). Loss-of-function *ELOC* mutations disrupt the Elongin C-pVHL interface, impairing HIF-α recruitment and subsequent ubiquitination, leading to HIF-α stabilization. The stabilized HIF-α heterodimerizes with HIF-β and translocates to the nucleus, where it drives transcription of oncogenic target genes, ultimately promoting tumorigenesis (3).

RCC ranks among the ten most common malignancies worldwide, with ccRCC being its predominant histologic subtype (4). *ELOC* (*TCEB1*)-mutant RCC, characterized by clear cell morphology and *ELOC* mutations, is a newly recognized molecular subtype first reported by Sato et al. in 2013 and subsequently formally included in the 2022 WHO classification (5, 6). Recent studies indicate that classic *ELOC*-inactivated RCC lacks *TSC1*, *TSC2*, or *mTOR* mutations (7), distinguishing it from RCC with fibromyomatous stroma (RCC-FMS) and reinforcing its status as a distinct entity.

Morphological and immunohistochemical features of *ELOC* (*TCEB1*)-mutant RCC frequently overlap with those of ccRCC, clear cell papillary renal cell tumor (ccPRCT), and RCC-FMS. Despite its mostly indolent clinical course, earlier TNM stage distribution, and favorable prognosis (8), these overlapping features make misdiagnosis common. Accurate molecular diagnosis is therefore crucial to avoid unnecessary adjuvant therapy and skewed clinical trial data.

Reported *ELOC* mutations in this subtype include *Y79C/L/S/N, E92K, A106D*, and C112Vfs*3, all located within or near the VHL-binding domain of the *ELOC*. *Y79* represents the predominant hotspot. While previous reports have documented individual cases with *E92K* and *A106D* variants (9–11), our study identifies a novel p.Y76C variant, predicted to disrupt the ELOC–pVHL interaction and exert a pathogenic effect. A recent large case series by Hou et al. (2026) further expanded the clinical and molecular spectrum of ELOC-mutant RCC (12).

## 2 Case report

### 2.1 Clinical presentation

A 52-year-old male was admitted in March 2025 after the incidental discovery of a left renal mass on routine ultrasound examination. He denied any associated symptoms, including lumbar pain, chills, fever, urinary urgency, dysuria, or gross hematuria. His surgical history included percutaneous endoscopic lumbar discectomy in 2017, with no other notable medical history. There was no family history of renal masses or hereditary renal cancer syndromes.

Physical examination revealed a soft, non-distended abdomen with no palpable masses, abdominal wall varices, tenderness, or costovertebral angle percussion tenderness. All routine laboratory findings were unremarkable.

Contrast-enhanced abdominal CT performed on March 14, 2025 showed both kidneys to be normal in size and morphology. Multiple bilateral small, well-defined, non-enhancing cystic lesions were noted. In the left kidney, a round, slightly hypodense lesion measuring 14×15 mm was observed, with a pre-contrast attenuation of ~35 HU. The lesion demonstrated intense enhancement in the arterial phase (~130 HU) and appeared hypoenhancing relative to the renal parenchyma in the delayed phase. The mass was located in the left kidney; no invasion of the adrenal gland, renal vein, or renal sinus was identified. No extrarenal masses or suspected metastases were seen on initial imaging. Imaging differential diagnosis included bilateral renal small cysts and an indeterminate left renal nodule, with malignancy not excluded (**Figures 1A–C**).

**Figure 1:**
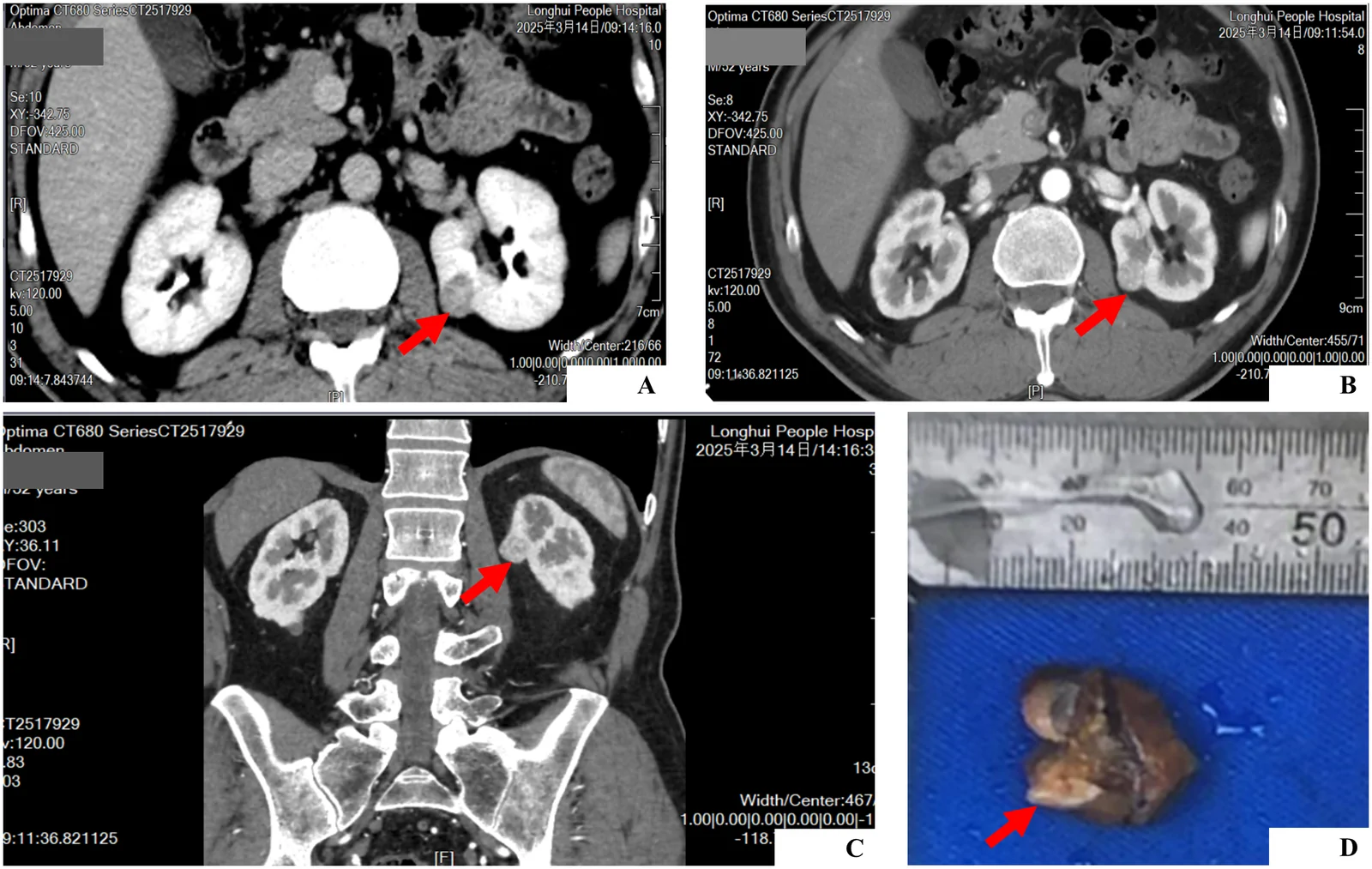
**(A, B, C)** Contrast-enhanced abdominal CT showing a round, slightly hypodense lesion measuring 14×15 mm was observed, with a pre-contrast attenuation of ~35 HU. The lesion demonstrated intense enhancement in the arterial phase (~130 HU) and appeared hypoenhancing relative to the renal parenchyma in the delayed phase. Differential diagnoses included bilateral renal small cysts and an indeterminate left renal nodule, with malignancy not excluded. **(D)** Gross examination revealed a well-circumscribed tumor measuring 1.5×1.0 cm.

### 2.2 Pathological findings

Gross examination revealed a well-circumscribed tumor measuring 1.5×1.0 cm (**Figure 1D**). The specimen was a retroperitoneal laparoscopic partial left nephrectomy. The specimen was received in its entirety, and representative sections were taken from the tumor and adjacent non-neoplastic parenchyma. Surgical margins were negative. No perirenal fat, renal sinus, or lymphovascular invasion was identified.

Microscopically, the tumor exhibited a nodular architecture partitioned by smooth muscle bundles, with branching tubular and papillary formations. The neoplastic cells were round to oval, with well-defined cell borders, clear cytoplasm, distinct nuclear membranes, and visible nucleoli. Mitotic figures were rare, and no conspicuous necrosis was identified (**Figures 2A–F**). Eosinophilic hyaline globules were not prominent in the present case and were not identified in the available sections.

**Figure 2:**
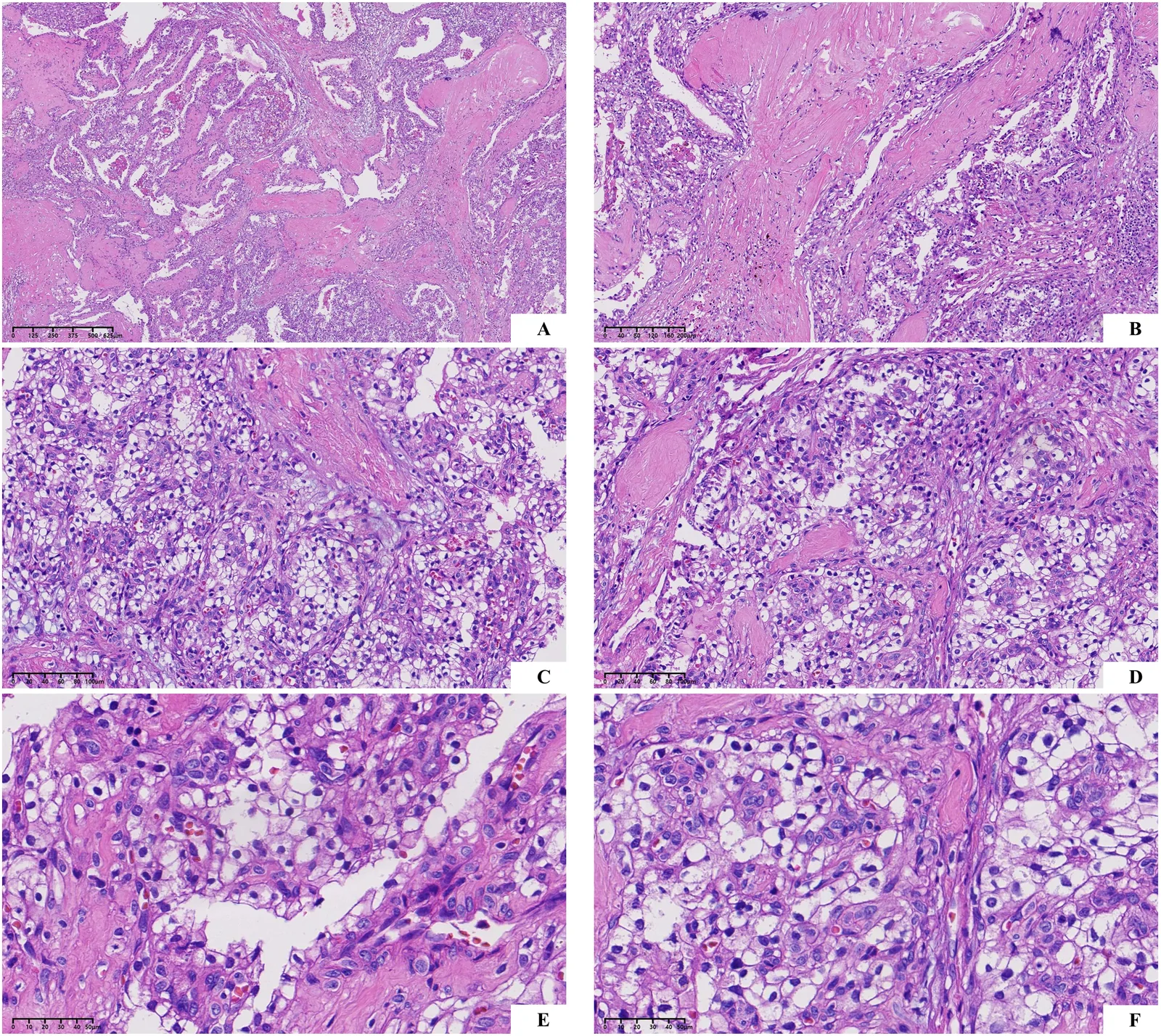
Microscopic features of *ELOC (TCEB1)-*RCC. The tumor exhibits a nodular architecture partitioned by smooth muscle bundles. The tumor cells show heterogeneous growth, predominantly forming branching tubular and papillary structures supported by delicate fibrovascular cores. The cells are columnar, arranged in a monolayer lining cystic spaces, with well-defined borders and abundant clear cytoplasm. Eosinophilic hyaline globules were not prominent in this case **(A–F)**.

Immunohistochemically, the tumor cells diffusely expressed cytokeratin (CK), high-molecular-weight cytokeratin (HMWCK), CK7, and CD10 (**Figures 3A–D**), carbonic anhydrase IX (CAIX), paired box 8 (PAX8), and α-methylacyl-CoA racemase (AMACR/P504S) (**Figures 3E–G**); P504S was diffuse but weak. The Ki-67 proliferation index was approximately 3% (**Figure 3H**); vimentin and smooth muscle actin (SMA) were negative in tumor cells and positive in adjacent stroma (**Figures 3I, J**). Transcription factor E3 (TFE3) showed partial weak positivity (**Figure 3K**), whereas CD117 (**Figure 3L**), HMB45, and Melan-A were negative. p53 exhibited weak-to-moderate staining in roughly 40% of tumor cell nuclei, suggestive of wild-type expression. Fluorescence *in situ* hybridization (FISH) detected no TFE3 gene rearrangement (data not shown).

**Figure 3:**
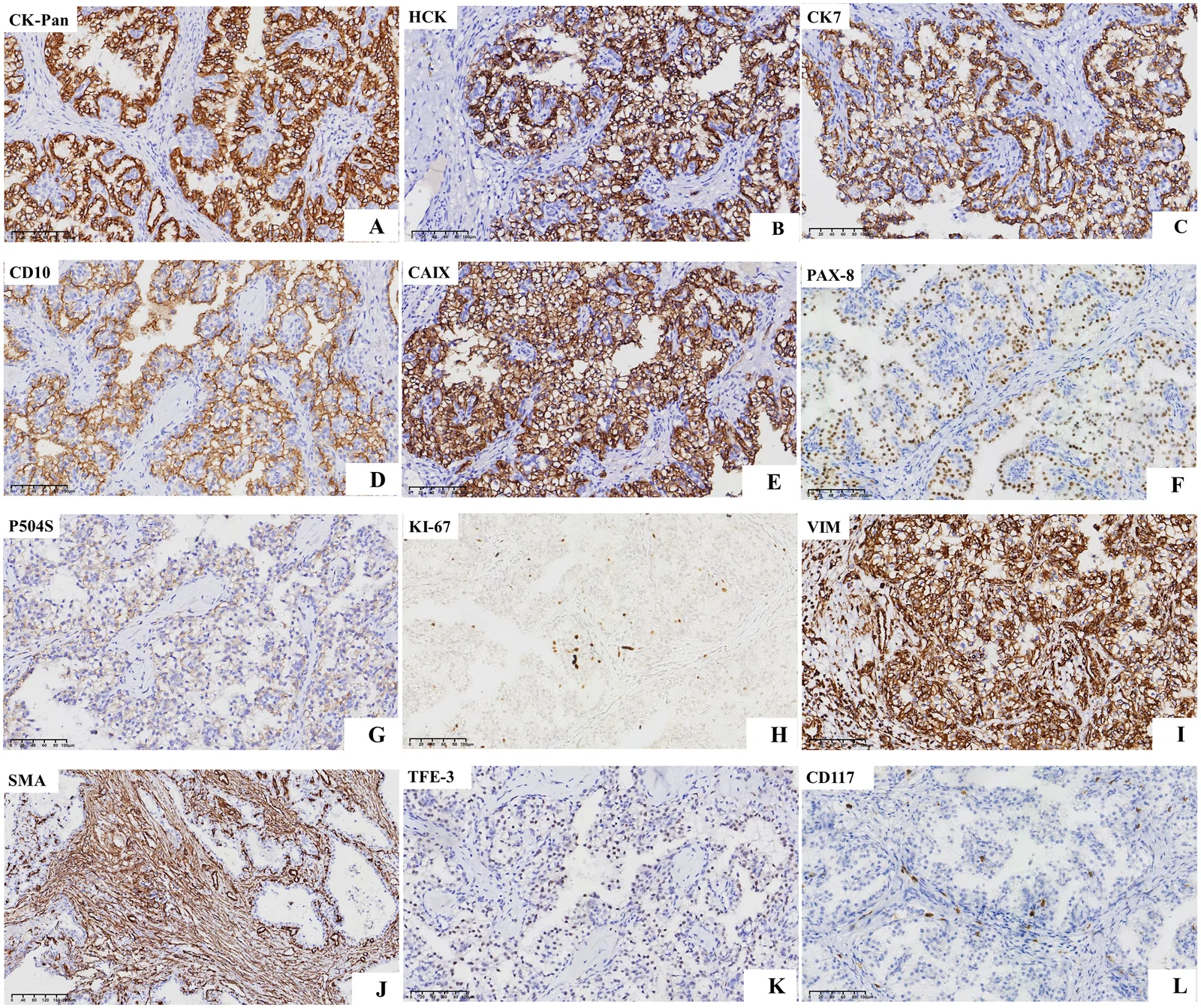
Immunohistochemical profile. The tumor cells diffusely expressed CK, HMWCK, CK7, and CD10 **(A–D)**, CAIX, PAX8, and P504S **(E–G)**; the Ki-67 proliferation index was approximately 3% **(H)**; vimentin and SMA were negative in tumor cells and positive in adjacent stroma **(I, J)**. TFE3 showed partial weak positivity **(K)**, whereas CD117 was negative **(L)**.

### 2.3 Genetic testing

Somatic variant analysis identified four altered loci. Variants in *TCEB1* (transcript NM_005648.3) p.N58Pfs*48 (c.171_172insCC), *TCEB1* p.Y76C (c.227A>G), and *ABL1* (transcript NM_005157.4) p.A857E (c.2570C>A) were classified as Category III, while *MCL1* (transcript NM_021960.4) amplification was designated as Category II. No germline variants were detected. Tumor mutational burden (TMB) was 0.0 mutations/Mb, and microsatellite stability (MSS) was confirmed. RNA-based fusion testing revealed no gene rearrangements or actionable genomic alterations. A final pathological diagnosis of ELOC (TCEB1)-mutant RCC was rendered. Pathological stage was pT1a; International Society of Urological Pathology (ISUP) nucleolar grade was Grade 1; no sarcomatoid or rhabdoid features were identified; no lymphovascular invasion, perirenal fat invasion, or renal sinus invasion was present; non-neoplastic renal parenchyma showed no remarkable changes (**Figure 4**).

**Figure 4:**
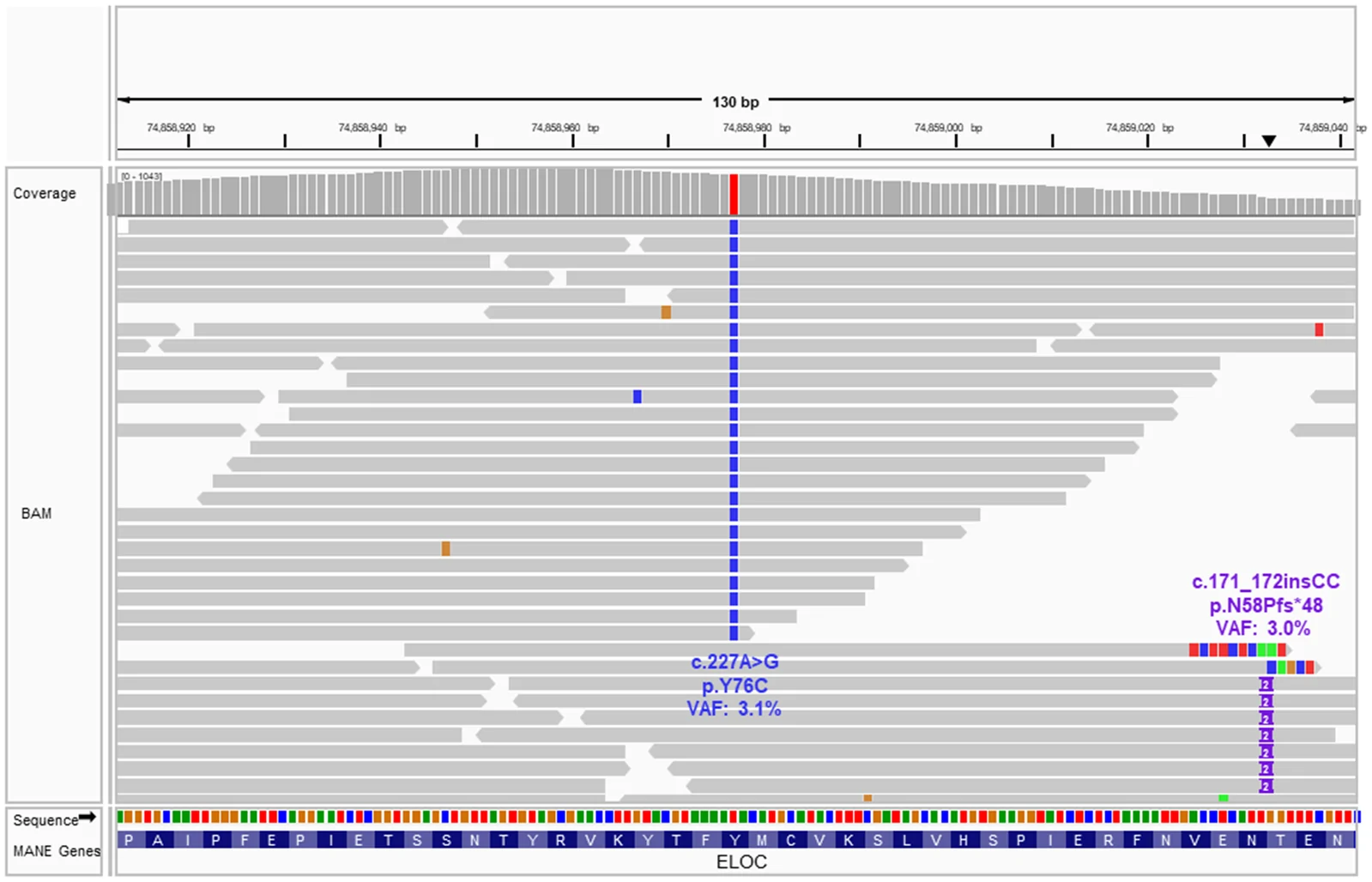
Integrative genomics viewer (IGV) screenshot showing two somatic *ELOC* (*TCEB1*) mutations in trans: a missense mutation c.227A>G (p.Y76C; variant allele frequency (VAF)=3.1%) and a frameshift insertion c.171_172insCC (p.N58Pfs*48; VAF = 3.0%). These mutations are observed on separate DNA molecules, confirming they reside on homologous chromosomes and are in a trans configuration.

### 2.4 Treatment and outcome

The patient underwent retroperitoneal laparoscopic partial left nephrectomy on March 16, 2025, without postoperative adjuvant therapy. The interval between initial imaging (March 14, 2025) and surgery was 2 days. Partial nephrectomy was chosen because the tumor was small and localized, and this approach preserves renal function; radiofrequency ablation was not considered because the lesion was indeterminate for malignancy and partial nephrectomy provided definitive pathology. He has remained alive and disease-free during follow-up, with no radiographic evidence of recurrence or metastasis. As of the last follow-up on August 20, 2026, 17 months after surgery, he remained disease-free; the last imaging was performed on August 16, 2026. A timeline of the clinical course is provided in **Supplementary Figure 1**.

## 3 Discussion

Epidemiological data from multiple cohorts indicate a distinct demographic profile for *ELOC (TCEB1)*-RCC. Among three cases from University Hospital Zurich, two were male (age range, 54–78 years; median, 69 years) (7). Of 13 patients in the UCLA and TCGA cohorts, 10 were male (range, 39–75 years; median, 59 years) (11). Among 15 patients from Ruijin Hospital Shanghai, 14 were male (range, 30–66 years; median, 55 years) (13). *ELOC* (*TCEB1*)-mutant RCC tends to occur in middle-aged to elderly males, consistent with our 52-year-old patient.

*ELOC* (*TCEB1*)*-*mutant RCC is a rare renal neoplasm with a mostly indolent clinical course, typically presenting at lower TNM stages and carrying a more favorable prognosis than ccRCC (8). Wang et al. reported that most *ELOC* (*TCEB1*)-RCC lesions present as solitary, mostly indolent tumors, and that patients with this subtype exhibit superior overall survival (OS) and progression-free survival (PFS) compared with stage- and grade- matched *VHL*-mutant ccRCC (11). In their cohort of 11 patients with low-stage disease, all remained disease-free over a median follow-up duration of 5.6 years, with only two deaths attributed to non-RCC-related causes (11). Our patient, who underwent partial nephrectomy without adjuvant therapy, has remained alive and recurrence-free for 17 months after surgery.

Microscopically, *ELOC* (*TCEB1*)-RCC exhibits a nodular architecture partitioned by smooth muscle bundles. The tumor cells show heterogeneous growth, predominantly forming branching tubular and papillary structures supported by delicate fibrovascular cores. The cells are columnar, arranged in a monolayer lining cystic spaces, with well-defined borders and abundant clear cytoplasm. Eosinophilic hyaline globules, resembling proteinaceous fluid in thyroid-like follicles, are frequently observed (13). However, they were not prominent in the present case.

Immunohistochemically, the combination of CK7, CAIX, and CD10 is useful in suggesting ELOC (TCEB1)-RCC, although none of these markers is entirely specific. CK7 positivity is critical for distinguishing *ELOC* (*TCEB1*)-RCC from low-grade ccRCC; however, it is also seen in ccPRCT and RCC-FMS, necessitating an integrated diagnostic approach (13, 14). CAIX positivity reflects HIF pathway impairment (15). Cup-shaped CAIX staining, though characteristic of ccPRCT, can also occur in some ccRCC cases, requiring careful morphological correlation (13, 16, 17). CD10 is positive in most ccRCC, *ELOC (TCEB1)*-RCC, and RCC-FMS, but negative in ccPRCT, aiding differential diagnosis (17, 18). GPNMB and P504S are reliable markers for RCC-FMS (13, 19–21). Additional markers (CD117, HMB45, Melan-A) and subtype-specific markers (TFE3, FH, SDHB, INI1/SMARCB1) help exclude other RCC subtypes. When morphological and IHC suggest an unusual profile without a clear molecular subtype, genetic testing should be promptly performed to guide accurate classification and prognostic stratification. In this context, Cheng et al. recently emphasized the diagnostic value of combined IHC and targeted sequencing in detecting rare *ELOC* variants (22). Similarly, Lin et al. proposed a practical algorithm integrating CK7, CAIX, and P504S to narrow the differential diagnosis before molecular confirmation (23).

*ELOC*, mapped to chromosome 8q21, encodes Elongin C, a component of the transcription factor B (SIII) complex involved in transcriptional elongation. Elongin C binds Elongin B, pVHL, Cullin2, and Rbx1 to form the VHL–Elongin B–Elongin C–Cullin2–Rbx1 (VCB-CR) complex, which possesses E3 ubiquitin ligase activity that targets HIFα for proteolytic degradation under normoxic conditions, thereby suppressing hypoxic signaling and tumorigenesis (24, 25). Mutational hotspots cluster within the Elongin C-pVHL binding interface, including Y79 (the most frequent site, with various substitutions), E92K, A100P, A106D, and C112Vfs*3 (5, 8, 10, 26, 27). The p.G92L variant, also located within this pocket, has been reported in Asian cohorts and similarly drives constitutive HIF activation and oncogenic transformation (13). These pathogenic variants are typically accompanied by secondary allelic loss, most often whole-chromosome 8 deletion (27–29). Canonical ccRCC drivers, including *PBRM1*, *SETD2*, *BAP1*, *TSC1*, *TSC2*, *mTOR*, *PIK3CA*, *PTEN*, and *ATM*, are absent in nearly all *ELOC*-RCC cases (11), reinforcing biallelic *ELOC* inactivation as the primary oncogenic driver and establishing this subtype as a distinct entity.

Our patient harbors a novel missense variant (p.Y76C) at the Elongin C-pVHL interface, which is absent from general population databases and predicted to be pathogenic by AlphaMissense, with a score of 0.9992 exceeding the recommended 0.564 threshold. This is accompanied by a frameshift mutation, p.N58Pfs*48, in trans. Beyond 8q deletions, MAP2K4 and HRAS amplifications are common secondary events in *ELOC* (*TCEB1*)-RCC (13, 28, 29); in our patient, we identified an ABL1 p.A857E variant (class III, allelic frequency ~2.6%, consistent with a subclonal passenger) and MCL1 amplification (class II, a potential cooperating modifier). Integrating morphology, IHC, and molecular genetics supports the diagnosis of *ELOC* (*TCEB1*)-mutant RCC in this case.

Most *ELOC* (*TCEB1*)-RCCs present at low pathological stage and nuclear grade (11, 13, 28). Surgical resection is generally curative for lesions lacking aggressive clinical features, and adjuvant therapy is not indicated for T1 to T2 tumors after radical or partial nephrectomy. In *ELOC* (*TCEB1*)-mutant RCC, *ELOC* mutations stabilize HIF-α, and targeting the HIF pathway or its downstream effectors may be rational. Unlike ccRCC, *ELOC*-mutant RCC usually lacks canonical VHL, PBRM1, SETD2, or BAP1 alterations; this may affect sensitivity to immune checkpoint inhibitors or tyrosine kinase inhibitors. However, clinical data remain limited, and advanced disease is currently treated according to recommendations for non-clear cell RCC subtypes (30). Recent reviews have emphasized that early genetic diagnosis can avoid overtreatment and guide surveillance intensity (22, 23).

Metastasis correlates with higher nuclear grade and advanced stage; high-grade lesions often acquire additional somatic aberrations in *TERT*, *PMS2*, or *NF1* alongside primary *ELOC* pathogenic variants (27). To date, only four stage III cases and two metastatic cases have been documented (27). *ELOC* (*TCEB1*)-mutant RCC usually follows a mostly indolent course with low metastatic potential.

Accurate pathological and molecular diagnosis can prevent unnecessary adjuvant therapy in surgically resected patients and avoid confounding results in clinical trials. Our patient, who underwent partial nephrectomy without adjuvant therapy, has maintained disease-free status, with no radiographic evidence of recurrence or metastasis. In summary, we report a rare case of *ELOC* (*TCEB1*)-mutant RCC in an adult patient harboring one novel missense variant (p.Y76C) and one frameshift variant (p.N58Pfs*48) in trans. The lesion also carries a subclonal *ABL1* p.A857E passenger alteration and an *MCL1* amplification that likely acts as a cooperating modifier. Histologically, the tumor shows nodular growth partitioned by smooth muscle bundles, with branching tubulopapillary structures; clear cytoplasm, rare mitoses, and no obvious necrosis. IHC revealed diffuse positivity for CK, HMWCK, CK7, CD10, CAIX, PAX8, and P504S; vimentin and SMA were negative in tumor cells and positive in adjacent stroma, with focal weak TFE-3 expression; FISH for *TFE3* rearrangement was negative. This integrated immunophenotypic profile, together with characteristic histomorphology and targeted NGS validation of *TCEB1* mutations, established a definitive diagnosis of *ELOC* (*TCEB1*)-mutant RCC, distinguishing it from mimics such as ccRCC, ccPRCT, and RCC-FMS. This integrating approach supports the diagnosis of *ELOC* (*TCEB1*)-mutant RCC in this case, helps avoid unnecessary postoperative adjuvant therapy, and optimizes long-term surveillance strategies for this indolent subtype.

## Data Availability

The original contributions presented in the study are included in the article/**Supplementary Material**. Further inquiries can be directed to the corresponding author.

## Supplementary material

Supplementary Figure 1 Timeline of the clinical course. An incidental left renal mass was found in March 2025. CT on March 14, 2025 showed a 14 × 15 mm left renal lesion without metastasis. Partial nephrectomy was performed on March 16, 2025. No adjuvant therapy was given; pathology confirmed *ELOC* (*TCEB1*)-mutant RCC, pT1a, ISUP grade 1. The last imaging was performed on August 16, 2026. As of the last follow-up on August 20, 2026, 17 months after surgery, he remained disease-free.
